# Recent advances in capsule-based dry powder inhaler technology

**DOI:** 10.1186/s40248-017-0092-5

**Published:** 2017-05-22

**Authors:** Federico Lavorini, Massimo Pistolesi, Omar S. Usmani

**Affiliations:** 1 Department of Experimental and Clinical Medicine, Careggi University Hospital, Largo Brambilla 3, 50134 Florence, Italy; 2 National Heart and Lung Institute, Imperial College London& Royal Brompton Hospital, London, UK

**Keywords:** Asthma, Chronic obstructive pulmonary disease, Drug delivery, Dry powder inhalers, Technology assessment

## Abstract

Pulmonary drug delivery is currently the focus of accelerated research and development because of the potential to produce maximum therapeutic benefit to patients by directly targeting drug to the site of pathology in the lungs. Among the available delivery options, the dry powder inhaler (DPI) is the preferred device for the treatment of an increasingly diverse range of diseases. However, because drug delivery from a DPI involves a complex interaction between the device and the patient, the engineering development of this medical technology is proving to be a great challenge. Development of DPI systems that target the delivery of fine drug particles to the deeper airways in the lungs using a combination of improved drug formulations and enhanced delivery device technologies means that each of these factors contributes to overall performance of the aerosol system. There are a large range of devices that are currently available, or under development, for clinical use, however no individual device shows superior clinical efficacy. A major concern that is very relevant in day-to-day clinical practice is the inter- and intra-patient variability of the drug dosage delivered to the deep lungs from the inhalation devices, where the extent of variability depends on the drug formulation, the device design, and the patient’s inhalation profile. This variability may result in under-dosing of drug to the patient and potential loss of pharmacological efficacy. This article reviews recent advances in capsule-based DPI technology and the introduction of the ‘disposable’ DPI device.

## Background

The benefits of inhaled therapy for the treatment of asthma and chronic obstructive pulmonary disease (COPD) have been recognised for many years. In comparison with oral or parenteral formulations, minute but therapeutic doses of drug are delivered topically into the airways where the active drug exerts its beneficial effects locally within the lungs. Unwanted systemic effects are minimised because the medication acts with maximum pulmonary specificity together with a rapid onset of action. As a result, aerosol formulations of bronchodilators and corticosteroids are the mainstay of modern treatment for asthma and COPD [1, 2]. Central to the success of inhaled treatment has been the availability of efficient aerosol delivery systems or inhalers.

Pressurised metered-dose inhalers (pMDIs) and dry powder inhalers (DPIs) are the devices most commonly used for drug delivery in the treatment of asthma and COPD. The pMDIs, which first became available in the mid-1950s, are globally the most widely-prescribed inhaler devices because they are cheap and use consistent technology to deliver a variety of medications. However, pMDIs do have some disadvantages in terms of effectiveness and usability. Most patients cannot use pMDIs correctly, even after repeated tuition [3], because pMDIs require good coordination between inhaler actuation and patient inspiration to ensure correct inhalation and deposition of drug in the lungs [4]. Misuse of pMDIs is frequent and associated with poorer asthma control in inhaled corticosteroid-treated asthma patients [5]. In contrast, because DPIs are actuated and driven by a patient’s inspiratory flow, they do not require propellants to generate the aerosol, nor coordination of inhaler actuation with inhalation [6]. However, a forceful and deep inhalation through the DPI is needed to de-aggregate the powder formulation into respirable particles as efficiently as possible in order to ensure that drug is delivered to the lungs [7, 8]. Although most patients are capable of generating enough flow to operate a DPI efficiently [9, 10], the need to inhale forcefully, and therefore generate a sufficient inspiratory flow, remains a problem for young children and patients with severe airflow limitation. For this reason, DPIs are not recommended for use in children under the age of 5 years. Less well known is that DPIs should also not be used in patients with compromised respiratory function who often do not have the inspiratory effort needed to ensure effective drug delivery from DPIs. At the same time that DPIs were introduced, there was an environmental concern that the chlorofluorocarbon (CFC) propellants used in pMDIs were contributing to irreparable damage to the ozone layer in the atmosphere. The pharmaceutical industry was, therefore, committed to the development of non-CFC propellants for use in pMDIs, and also to the development of DPIs that did not require any propellant at all [6–8]. Reformulation of pMDIs to change to hydrofluoroalkane propellants was challenging but resulted in drug formulations with favorable safety and tolerability profiles, although the need to reformulate inhaled corticosteroids (ICS) and long-acting beta-adrenergic bronchodilators (LABA) for use in pMDIs presented particular technical difficulties, especially regarding the achievement of dose-content uniformity. Another important distinction between DPIs and pMDIs, particularly those delivering standard coarse (>2 μm) aerosol particles, is that with the latter inhalers no more than 20% of the emitted dose reaches the lungs [6–8]. Conversely, DPIs have been associated with a pulmonary drug deposition rate that can be as high as 40% of the administered dose, provided patients use optimally-controlled inhalation flows through the device, otherwise lung deposition can be as low as ~15%. The high speed of the aerosol droplets exiting from a pMDI, or the high inhalation flows needed from some DPIs may result in marked droplet deposition in the oropharynx (between 50% and 80% of the administered dose), with subsequent potential for local adverse drug effects such as oral candidiasis and dysphonia, and systemic drug absorption after swallowing [11]. These issues may be resolved somewhat through the use of spacer devices in conjunction with the pMDI or the use of DPIs that require slower inhalation flows [12]. Bronchoconstriction is an uncommon adverse reaction following use of pMDIs, and is thought to be caused by excipients, such as oleic acid, possibly in combination with the propellant [13], whereas DPIs contain no propellants or preservatives [14].

**Fig. 1:**
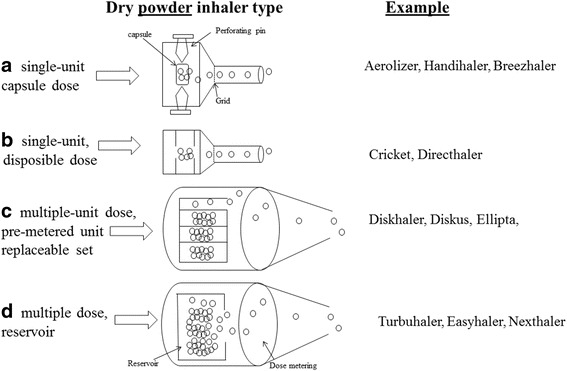
**a**-**d** types of dry powder inhalers and corresponding mechanisms for drug deagglomeration and dispersion. Modified from [8]

The main types of DPI systems are shown in Fig. 1. The single-unit dose inhaler requires the patient to load a single hard gelatine capsule containing the powder formulation into the device before each use (Fig. 1a). This is a very common type of DPI device currently available. Figure 1b shows a device containing a pre-metered amount of a single dose that is discarded after use. Multi-unit devices deliver individual doses from pre-metered replaceable blisters, disks, dimples or tubes (Fig. 1c). Multiple-dose reservoir inhalers (Fig. 1d) contain a bulk amount of drug powder in the device with a built in mechanism to meter a single dose and individual doses are delivered with each actuation. The multi-unit inhalers (Fig. 1c) are likely to ensure greater dosage control and chemical stability of the formulation than multiple dose types (Fig. 1d); however, the former are more expensive than the latter.

This brief review focuses on recent improvements in capsule-based DPI technology and on the recent introduction of capsule-based disposable inhalers.

### Capsule-based DPI technology

Although therapeutic application of capsule-based DPIs began in the middle of last century with the introduction of the Aerohaler® for the delivery of antibiotics,[15] the Spinhaler®, introduced at the end of the 1960s, was the first DPI containing a powder formulation of bronchoactive drugs in a gelatine capsule, which the patient loaded into the device prior to use. Since then, DPI systems have constantly evolved in technology and performance, a trend that still continues [16].

**Fig. 2:**
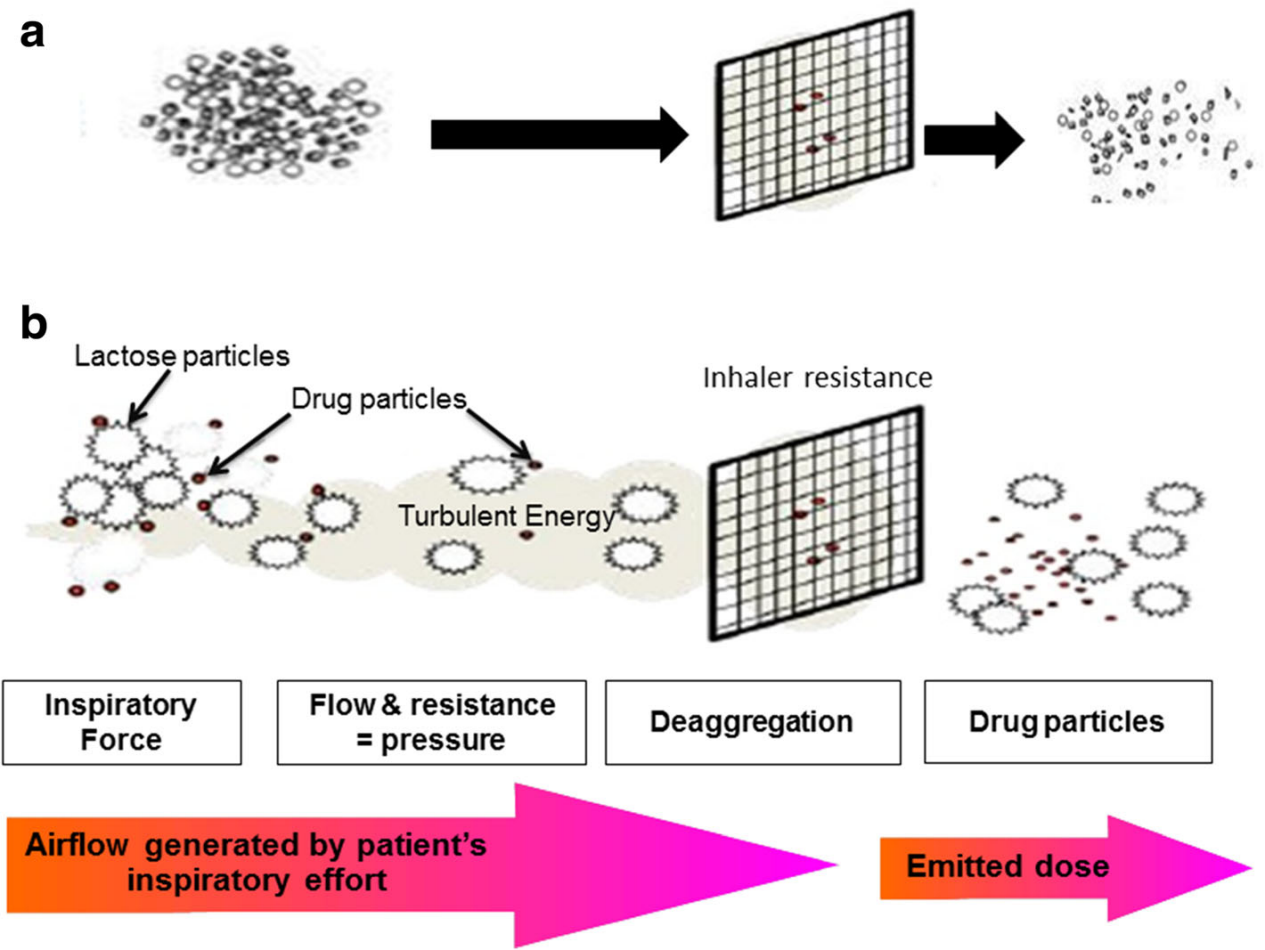
Schematic diagram of dry powder inhaler formulations and dispensing powder mechanisms. **a** Drug-only formulation (drug agglomerates); **b** Carrier-based formulation. See text for further details. Modified from [7, 8]

DPI formulations may either be fine powder drug (particle size <5 μm) blended with larger carrier particles (generally lactose) to prevent aggregation and increase powder flow prior to aerosolization, or it may consist of drug alone (Fig. 2). In all cases, the powder formulations travel along the airways to deposit in the targeted areas of the lung, and then dissolve to exert their pharmacodynamic effect or are absorbed to reach systemic targets. A drug particle size between 1 and 5 μm is needed for entry into the deep lung by inhalation and particles of 1–2 μm are most suitable for reaching the small airways (an important anatomical target for the treatment of asthma and COPD) and alveolar epithelium (an important target for systemic delivery/absorption of orally inhaled products) [17].

**Fig. 3:**
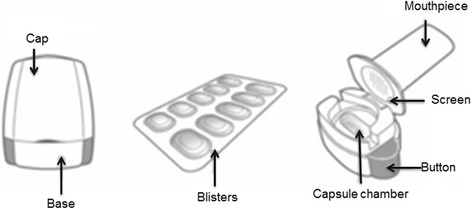
The Breezhaler capsule-based dry powder inhaler

The role of the technology in DPI devices is to disperse the powder mixtures into a respirable fine drug particle fraction by aerodynamic means. The aerodynamic behavior of a DPI is affected by its design, dimension, and geometry of the functional engineered device parts, such as the air-inlet/air-outlet, inhaler resistance, mechanisms of disaggregating powder mixtures (helicoids, sieves, cyclone channel) and emptying the dose (Venturi-effect, centrifugal forces, spinning/twisting). For instance, the air-inlet size has been shown to have a significant impact on powder dispersion at different inhalation flow rates by varying the inlet jet flow turbulences and particle interaction velocity [18, 19]. The performance of the device can also be modified by the resistance to airflow, which has a direct impact on the peak inspiratory flow (PIF), acceleration rate, inhaled volume to reach PIF, and total inhalation time [20].

Furthermore, the shape of the drug particle affects DPI performance. For instance, elongated particles have been found to achieve higher fine particle fractions released due to the unstable particle interactions [21]. Finally, and probably most importantly, interactions between drug and carrier particles are crucial with respect to the formulation performance [22]. Irregular surface structures prevent the particles from close interaction and ease the separation from each other upon aerodynamic stress.

Particle engineering using additional excipients, such as amino acids or sugar derivatives, in the drug-carrier formulations is another field of research to design the particle and surface characteristics for use in carrier-based and carrier-free DPI systems [23]. An example of this is a recently launched DPI formulation delivering tobramycin for the treatment of cystic fibrosis. This DPI formulation is characterized by microspheres of a submicron oil-in-water emulsion of tobramycin, distearoyl phosphatidylcholine and calcium chloride that is manufactured by spray drying to form porous particles of amorphous tobramycin. This novel capsule-based DPI formulation has been shown to achieve lung deposition of 34.2% of the delivered dose [24].

The performance of DPI formulations is also related to the characteristics of the primary packaging, from which the formulation is released upon activation in the device. Due to the ease and availability of capsule filling, DPIs use hard gelatin or hypromellose capsules as pre-metered monodose unit systems [25]. Capsule activation is achieved by shear-force opening, needle-piercing, or cutting of the capsules. Key characteristics of the capsule are the moisture content and water activity, puncturing performance in the device and powder retention [25]. The moisture content of the capsule depends on the environmental moisture level to which it equilibrates. Hypromellose capsules have lower the total water content than gelatin capsules (about 6% versus 14% water at 50% relative humidity [RH]); even so, the water activity between the shells remains similar when equilibrated to the same RH storage conditions. Puncturing by needles is the most frequent mechanism used by DPI systems to release drug powder from the capsules [25]. Generally, the number of needles used for puncturing ranges from 1 to 8 and puncturing can occur from the top of the capsule or from the side. Although the puncturing behavior under normal storage conditions has been found to be good for both types of capsules, hypromellose capsules exhibit high performance upon puncturing even under very dry conditions without any fractions or particles from the shell [25].

The quantity of drug powder retention in the capsule depends on the interplay between the surface properties of the capsule shell, the characteristics of the interactive powder mixture, and the functioning of the device. With a given formulation and device, powder retention can be modified by changing the surface characteristics of the capsule to achieve the optimal performance target [25]. This performance target might well be the emitted fine particle fraction, which is also based on the de-agglomeration effect of the capsule on the powder mixture [25].

All currently available passive DPI systems rely solely on the inspiratory force of the patient to disperse drug powders. When the patient activates the DPI and inhales, airflow through the device creates shear and turbulence; air is introduced into the powder bed which is fluidized and enters the patient’s airways. Thus, the drug particles, separated from the carrier particles, are carried deep into the lungs, while the larger carrier particles impact in the oropharynx and are cleared. As a result, deposition into the lungs is determined by the patient’s variable inspiratory airflow [8]. However, each DPI system has a specific airflow resistance that is due to the physical design of the device; this means that a threshold inspiratory force is required to achieve the correct flow rate to aerosolize, deagglomerate, and disperse the powder formulation in order to achieve an effective therapeutic response. It is the physical design of the DPI that establishes its specific resistance to airflow (measured as the square root of the pressure drop across the device divided by the flow rate through the device), with current designs having specific resistance values ranging from about 0.05 to 0.3 cmH_2_O/L/min [26]. To produce a fine powder aerosol with increased delivery to the lung, a DPI characterized as having a low resistance requires an inspiratory flow of >90 L/min, a medium-resistance DPI requires 50-60 L/min, and a high-resistance DPI requires <50 L/min [26]. Of note, DPIs with high intrinsic resistance, and hence increased pressure drop across the device, tend to produce a greater lung deposition than those with low intrinsic resistance;[26] however, the clinical significance of this is unknown.

### Breezhaler^®^: an example of recent capsule-based DPI

The Breezhaler device (Fig. 3) is a single-dose DPI based on the Aerolizer technology with design changes intended to improve device handling and appearance. The Breezhaler is used to deliver drug from capsules containing the long-acting beta-adrenergic bronchodilator indacaterol maleate and the long-acting muscarinic antagonist glycopyrronium. The device has been designed to have lower internal airflow resistance (0.15 cmH_2_O/L/min) than that of other capsule–based DPIs such as the HandiHaler device (0.22 cmH_2_O/L/min). Due to its low intrinsic internal resistance it requires high inspiratory flow rates (100 and 117 L/min) to obtain a mean pressure drop of 4 kPa within the device [27]. However, Pavkov et al. [28] and Singh et al. [29] have shown that COPD patients are able to generate a peak inspiratory airflow of approximately 90 L/min through the Breezhaler device, overcoming a pressure drop of approximately 3 kPa. Although performance of DPIs are usually compared at 4 kPa pressure drop [26], studies in COPD patients demonstrated consistent dose delivery from Breezhaler using flow rates between 50 and 100 L/min [28, 30] corresponding to a pressure drop less than 4 kPa. DPIs with low resistance tend to be more accepted by patients than those with high resistance [31]. In elderly patients, the ability to generate sufficient inspiratory flow through a DPI is compromised, irrespective of the presence of airway obstruction, as shown by Janssens et al.[32] who demonstrated that 30, 20 and 12.5% of an elderly population were not able to reach the minimum peak inspiratory flow of 45 L/min when using the Turbuhaler, Diskus and Aerolizer DPIs, respectively. Keeping this in mind, the choice of a lower resistance DPI, which is relatively insensitive to changes in peak inspiratory flow at lower flow levels, would definitely benefit the patients. The recent ERS/ISAM taskforce on inhalers [33] recommends patients “to inhale forcefully from the beginning of inspiration, as deeply as possible, and to continue to inhale for as long as possible”. This is because, with a DPI, inhalation should be forceful enough to disperse the micronized drug from the lactose-based carrier into a fine particle dose. However, it is not the absolute inspiratory flow that determines the fine particle dose from an inhaler, rather it is the turbulent energy obtained from the relationship between patient’s inhalation and inhaler device resistance.

High air velocities *within* the inhaler are required for effective dispersion rather than high airflow *through* the inhaler. The higher the airflow, the higher the powder dispersion generating a fine particulate, even if such a high airflow leads to a higher impaction losses in the proximal airways and, as a result, to a lower dose reaching peripheral airways [27, 34]. On the other hand, a lower airflow contributes to deeper lung deposition of the powdered drug, even if a too low airflow (such as that occurring in patients with the worst disease severity) can limit deposition by affecting powder disaggregation and dispersion [27, 34]. Furthermore, when using a single-dose DPI, such as the Breezhaler, it is also recommended that patients are instructed to perform two separate inhalations for each dose [35].

Ease of use and the presence of reliable feedback is considered by the majority of patients as the most important features of an inhaler. Patients are in general not assured that they have taken the full dose correctly with most inhalation devices and want ease of device use to increase their acceptance of the device. Healthcare practitioners emphasize an increase in patient satisfaction because this potentially increases adherence. To properly operate the Breezhaler device, patients must load the device by inserting the capsule of powder into a chamber [35]. After closing the lid, the capsule is perforated with needles fixed to pressure buttons; the patient then inhales through the device, which causes the capsule to rotate within the device chamber, and this creates a distinctive ‘whirring’ noise as the capsule spins. Furthermore, patients can see that they have inhaled all the powder because the capsule is clear. It is clear when the device is empty and needs to be refilled. Thus, the Breezhaler device has three potential feedback mechanisms that give patients an indication that the full medication dose has been delivered: (a) the device gives off a ‘whirring noise’ as the capsule spins each time a patient inhales (providing positive auditory feedback that the medication is being released from the capsule); (b) the medication formulation contains lactose, a small amount of which stays in the patient’s mouth during inhalation and most patients can taste it; (c) the transparent capsule will be visibly empty or have only residual traces of powder after successful inhalation, giving visual feedback to the patient that the drug has been released [35].

### Limitations of capsule-based DPI

#### Handling-related limitations

Capsule-based DPI requires that single doses are individually loaded into the inhaler immediately before use: this is inconvenient for some patients and does not allow direct dose counting. The device then needs to be primed by breaking the capsule, and then, depending on the patient’s breathing profile, the inhalation process must be continued or repeated until the capsule is emptied: this may result in under-dosing and high dose variability. In addition, the sequence of steps required to properly load the device may not be easy for children or elderly patients with diminished dexterity. It has been shown that patients were more confident of the medication being taken correctly when using the Breezhaler, and found it more comfortable and simpler to use compared with the Handihaler [36]; however, the methodology used to investigate preference for and satisfaction with a particular inhaler was not validated, and preference was assessed over a limited time frame.

#### Technical limitations

It has been shown that the air inlet size and grid structure of the Aerolizer capsule-based DPI were found to impact significantly on aerosolization of the carrier-based powder [37]. More specifically, the powder contained in the capsule needs to be released through the pierced holes at each end of the capsule during aerosolization. The centrifugal force generated by the spinning capsule will eject the powder through the capsule holes. When the air flow increases, the capsule rotation speed will increase significantly,[37] which increases the centrifugal force hence facilitating the powder exit. As shown with the Aerolizer, capsule retention has an impact on the fine particle fraction (FPF) and, more importantly, affects the dose delivered to patients. In addition, the FPF is higher when the airflow rate is increased [26]. Thus, at a flow rate <30 L/min, greater variations in drug retained in the capsules can be observed compared with higher flow rates.

### Disposable capsule-based DPI

In inhalation medicine, from an economic perspective, the trend of the past two decades indisputably has been the introduction of a large number of generic devices for administration of ICS and beta2-agonists for the treatment of asthma and COPD [38]. In many countries, this development and the pressure on healthcare budgets have resulted in a significant switch from branded to generic medications and devices. The chronic nature of these asthma and COPD requires a lifetime of treatment, with a high frequency of drug administration and, therefore, high costs. In addition, many new applications of inhaled therapy (e.g. vaccination, rescue medication, enzymes, peptides) may require inhaler specifications that cannot be achieved using classic inhaler technology and for several of these applications, disposable inhaler versions maybe preferred. By design, capsule-based DPIs seem most suitable as disposables and can also be developed as single-use devices. Pharmaceutical companies using these devices have to adjust their formulation to the performance of the inhaler, which often includes the incorporation of new excipients, up to substantial amounts, and the use of complex preparation techniques. An example is the PulmoSphere® formulation for the antibiotic tobramycin in the former Turbospin capsule-based inhaler, now referred to as Podhaler for tobramycin. However, disposable DPIs used in these settings still need to be simple and inexpensive, but also highly effective and reproducible. Many recipients in, for instance, vaccination programs will be inhaler-naive and providers may not always be well-trained. Simple design will therefore facilitate correct use, preferably almost by intuition.

## Conclusions

In the last decade, we have observed an increasing interest in DPI technology in response to the need for alternatives to ozone-depleting and greenhouse gas-propelled inhaler devices, and new approaches to the delivery of potent drugs of biological origin. The characteristics and dynamic interaction between fine drug particles and carrier particles, as well as the interaction between particles and inhaler characteristics, have now been elucidated [39]. The future development of DPI devices will have to focus on simplicity of use, reliability, consistency, suitability for a large range of products and doses, feedback mechanisms to the patients, and last but not least, cost effectiveness. For pulmonary drug delivery, the device–patient interface has been shown to be of critical importance because patients vary in their ability to use inhalation products, but also they differ in the level of education required for appropriate use. Although existing DPIs are efficient devices for delivering drugs to the lung, there is substantial room for improvements without losing the cost-effectiveness advantages. As a greater understanding of powdered drug properties and their influence on performance is obtained, it will be possible to adopt sophisticated technological approaches to solve the problems associated with efficient drug delivery to achieve local and systemic pharmacologic effect.

## Abbreviations

CFC: Chlorofluorocarbon
COPD: Chronic obstructive pulmonary disease
DPI: Dry powder inhaler
FPF: Fine particle fraction
ICS: Inhaled corticosteroids
LABA: Long-acting beta-adrenergic bronchodilators
PIF: Peak inspiratory flow
pMDIs: Pressurised metered-dose inhalers
RH: Relative humidity
