# Erratum to: Recent advances in capsule-based dry powder inhaler technology

**DOI:** 10.1186/s40248-017-0100-9

**Published:** 2017-06-23

**Authors:** Federico Lavorini, Massimo Pistolesi, Omar S. Usmani

**Affiliations:** 1 Department of Experimental and Clinical Medicine, Careggi University Hospital, Florence, Italy; 2 National Heart and Lung Institute, Imperial College London& Royal Brompton Hospital, London, UK

## Erratum

After publication of the article [1] it was brought to our attention that resistance values of Breezahler (0.15 cmH2O/L/min) and Handihaler (0.22 cmH2O/L/min) are incorrect. (page 4 right column, lines 14 and 16 from below). The correct resistance values of Breezhaler and Handihaler are 0.07 and 0.16 cmH2O/L.min^-1^, respectively. We apologise for the inaccuracy.
